# The effect of oxygen pickup during selective laser melting on the microstructure and mechanical properties of Ti–6Al–4V lattices

**DOI:** 10.1016/j.heliyon.2019.e02813

**Published:** 2019-12-05

**Authors:** M. Velasco-Castro, E. Hernández-Nava, I.A. Figueroa, I. Todd, R. Goodall

**Affiliations:** a Instituto de Investigaciones en Materiales, Universidad Nacional Autónoma de México, Circuito Exterior S/N, Cd. Universitaria, México, DF, C.P. 04510, Mexico; b Department of Material Science & Engineering, The University of Sheffield, Sir Robert Hadfield Building, Mappin St, Sheffield, S13 JD, UK

**Keywords:** Materials science, Mechanical properties, Cellular materials, Additive manufacturing, Titanium alloys

## Abstract

Additive manufacturing techniques such as Selective Laser Melting (SLM) can produce complex shapes with relatively thin sections and fine detail. However, common materials for the process, such as Ti–6Al–4V, have microstructure and properties that are sensitive to the pickup of interstitial impurities, such as oxygen, which the material will be exposed to during the process. This problem would be especially severe for parts with thin sections, where surface effects can be more significant, and where poor properties may coincide with locally-elevated stress. Here we explore the effects of oxygen level in thin sections with the use of lattice materials (materials which can be considered to consist exclusively of near-surface material). Oxygen levels are artificially raised using repeated melting passes to result in more pickup, leading to significantly reduced ductility and hence reduced strength measured in compression. A ductile to brittle transition in strut failure mechanism is found with increasing number of melting passes, with significant modification in chemistry and crystallographic structure, despite the presence of a similar fine plate-like microstructure throughout.

## 1 Introduction

Additive Manufacturing (AM) technologies have demonstrated their capabilities for processing a wide range of materials in terms of freeform design. Within AM focussed on metals, Selective Electron Beam Melting (SEBM) and Selective Laser Melting (SLM) are among the most frequently used techniques. The advantages and disadvantages of these two technologies have been extensively discussed [1, 2]. The SLM route presents certain advantages, such as where surface finish is a key requirement, due to the use of thinner layers and smaller particle sizes. These factors, combined with the ability to focus the laser onto a small spot suggest that SLM can manufacture components with finer detail and thinner sections. However, very thin depositions consisting of a few layers only, and the first layers in overhangs (material built over powder, frequently requiring anchor/support structures) may suffer from a lack of integrity due to the beam energy being too great for the number of layers deposited; work [3] has shown such effect on angled truss structures. Although commercial systems apply their own algorithms to modify beam speed and power in areas where the thickness of the part tends towards dimensions which would cause problems, due to commercial restrictions most of these functions are not released to the public domain [4]. Properties and structures of materials that have suffered excess of beam power in AM, in some cases achieving a highly oxidised state (“discoloured” areas) are not reported in the literature, leaving their mechanical performance, and the potential for harm from such problems, unknown. Bulk material properties of such sections may be difficult to find in the literature compromising performance and simulation models which may be used in design for applications. In this study, the mechanical properties and microstructure of thin-walled materials repeatedly exposed to the heat source during each layer of the build are assessed, as an attempt to mimic conditions of excessive energy input leading to oxidation (as the time spent molten is greater). Diamond-like lattice structures are fabricated, as a means to access thin-walled materials in a bulk form suitable for mechanical assessment. These also represent an example of a material type for which AM presents advantages over other manufacturing techniques. In processing these lattices by SLM, repeated beam passes are used in each layer to increase energy input in order to cause the embrittlement, possible oxidation and characteristic discolouration of thin sections. Mechanical properties are reported as well as microstructure and chemical composition.

## 2 Experimental procedure

The AM process was carried out in an SLM 250 Renishaw system using Magics build processor to configure the layer build file. Overall, default beam parameters for a 30 μm process were used, where a beam power of 200 W, a point distance of 75 μm, a hatch spacing of 65 μm and an exposure time of 50 μs were applied in a rastering-like pattern rotating 67° in each layer. Pre-alloyed Ti–6Al–4V powder with a particle size of 20–45μm was employed for manufacturing 12 diamond-like lattices, three for each of four conditions; 1, 3, 5 and 9 beam passes in each layer. This was used as a means to vary the total energy input. Before the process starts, the Renishaw system preheats the build platform to reach 170°C while degassing to the build chamber. Once the oxygen sensors register that the oxygen level has dropped to 1000ppm, an argon atmosphere, which is continuously recirculated during the process, is allowed in. The recirculation cycle is from the top to one side of the chamber, and continues until the build is completed. Once processed, all samples were stress relieved by a heat treatment under vacuum. The thermal cycle was composed of a 350°C hold for 1hr and a soak time of 0.5hrs then a further increase to 850°C with a hold time of 1hr, before finally furnace cooling. All samples included a solid square base for easy cut-off from the process substrate by Electro-Discharge Machining (EDM) after the heat treatments.

Compression testing took place in a Zwick/Roell Z050 test rig machine with a load cell of maximum capacity of 50 kN at a strain rate of 3.3×10^−3^ s^−1^. Each test used ceramic plates in contact with the sample to minimise friction forces. Values of failure strength were obtained from the linear part of the stress-strain curves up to point of 0.1% once converted from load and displacement. Maximum stress is also reported from the peak load, see table 1. Once tested to failure, samples were prepared for microhardness measurements in a Struers Durascan 70 automated indenter. All samples were indented under 0.1 kg load for a dwell time of 5 s, with indents spaced by 0.5 mm. Microscopic analysis included scanning electron microscopy (SEM) with energy dispersive X-ray spectroscopy (EDX), using a FEI - Inspect F microscope with accelerating voltage of 15kV, a working distance of 10mm with a 3 μm spot size. Details of the micropreparation steps can be found elsewhere [5]. Additionally, high resolution imaging was carried out in a Schottky Field Emission, Transmission Electron Microscope JEOL JEM-ARM200F with aberration corrector in STEM mode. The specimens were micro machined by focused ion beam using a JEM-9320. X-ray diffraction (XRD) was carried out using a Siemens D5000 diffractometer with Cu-Kα radiation. Finally, chemical composition of all materials was assessed by X-ray fluorescence/Inductively Coupled Plasma (XRF/ICP) for aluminium and gas combustion LECO analysis for oxygen and nitrogen. In addition to samples from built parts, "discoloured" powder, which has been exposed to elevated temperature but then been ejected from the build envelope due to the flow of recirculated argon gas, was chemically assessed.

**Table 1:** Relative density and mechanical properties of all samples. LBP stands for laser beam pass. Chemical composition of is given in wt%. Uncertainties for Al, V and Ti detected by ICP are: 1 ppm of 30%, 10 ppm of 20%, 100 ppm of 10%, 0.10–1% of 5%, 1.1–2% of 3%, 2.1–10% of 2%, 11–30% of 1%, 40% of 2% and 50–100% of 2–5%. Other elements found were: Cu, Si, and Fe showing a fairly low composition of 0.1–0.2, 0.15–0.16 and 0.01–0.02 respectively.

Property	Sample			
	1LBP	3LBP	5LBP	9LBP
Rel. dens	0.102 ± 0.003	0.149 ± 0.004	0.184 ± 0.002	0.246 ± 0.005
Failure Stress (MPa)	12.1 ± 0.7	5.6 ± 0.2	3.2 ± 1	4.4 ± 0.8
Maximum stress (MPa)	12.6 ± 2.6	6.4 ± 1.4	5.5 ± 0.5	4.7 ± 2.0
Elongation to failure (%)	4.7 ± 0.2	1.8 ± 0.2	1.8 ± 0.2	1.06 ± 0.2
HV	424 ± 25	494 ± 33	567 ± 42	557 ± 41
Al	6.13	6.07	5.97	5.54
V	4.07	4.14	4.13	4.27
O	0.412 ± 0.0076	0.642 ± 0.0076	0.642 ± 0.0076	1.15 ± 0.0076
N	0.132 ± 0.0030	0.217 ± 0.0030	0.214 ± 0.0030	0.319 ± 0.0030
C	0.081 ± 0.012	0.026 ± 0.012	0.026 ± 0.012	0.026 ± 0.012
Ti	89.39	88.40	87.92	88.33
Oxygen in “discoloured” powder	1.30 ± 0.0076			

## 3 Results

While relative density was measured prior to compression tests, SEM imaging, chemical analysis by XRF/LECO and Vickers micro hardness (HV) measurements were obtained from tested specimens. Results are summarised in table 1.

### 3.1 Additive manufacturing results

**Fig. 1:**
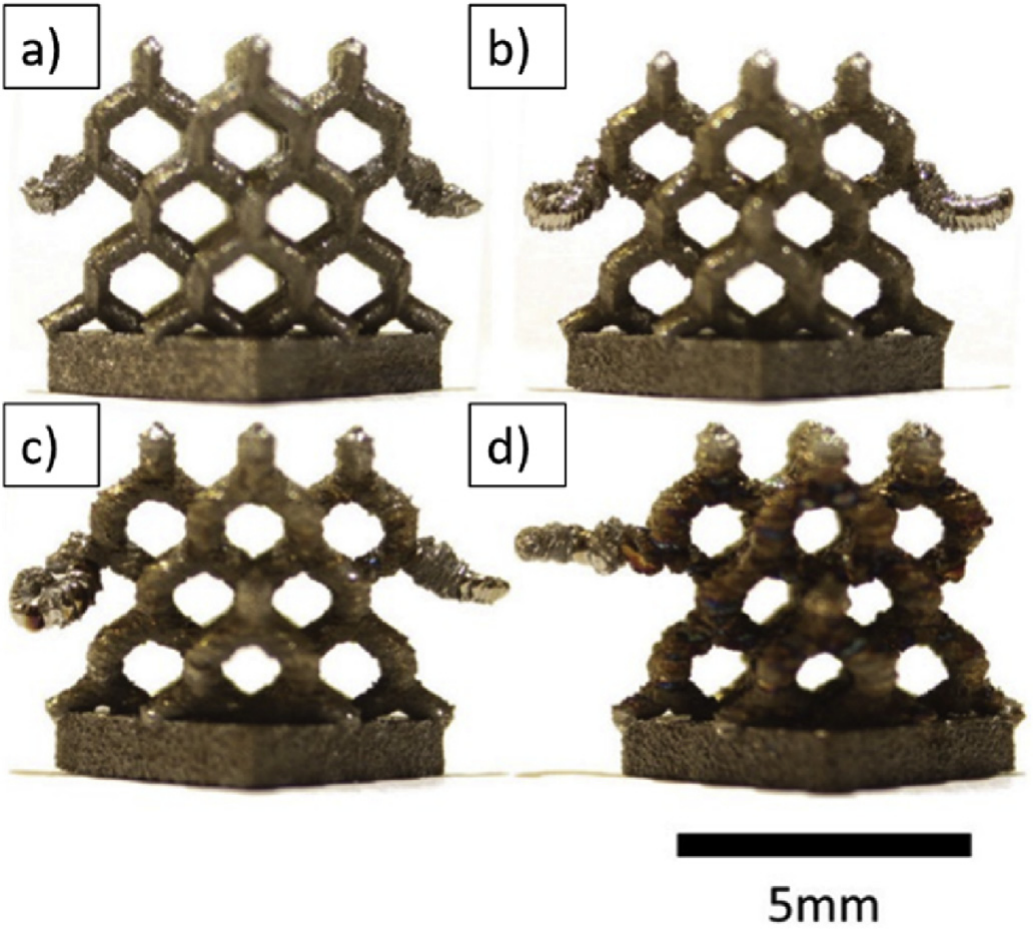
Diamond-like lattice samples after manufacture, heat treatment and removal from the substrate. One example is shown for each condition, (a) 0.102, (b) 0.149, (c) 0.184 and (d) 0.246 relative density.

Examples of processed samples are shown in Fig. 1. A relative increase in the size of the lattice struts is accompanied by a change in colouration, suggesting that the repetitive heat passes are responsible for this change. Measurements of relative density, table 1, show that density increases with the number of passes. It can also be noticed that truss shape specified in the design was compromised, especially around unconnected trusses. This change in shape could be due to an excess of heat, as the unsupported area was exposed with no structure for heat dissipation. The effect of such struts on the response to a compressive load may not be significant though as these trusses are not connected in such a way that they will be loaded. Results of oxygen content analysis is well correlated with the change in colouration, and the number of laser passes, see table 1. While samples with only one pass show the minimum content of interstitial impurities, samples processed with the maximum number of passes show a higher content.

### 3.2 Compression testing

**Fig. 2:**
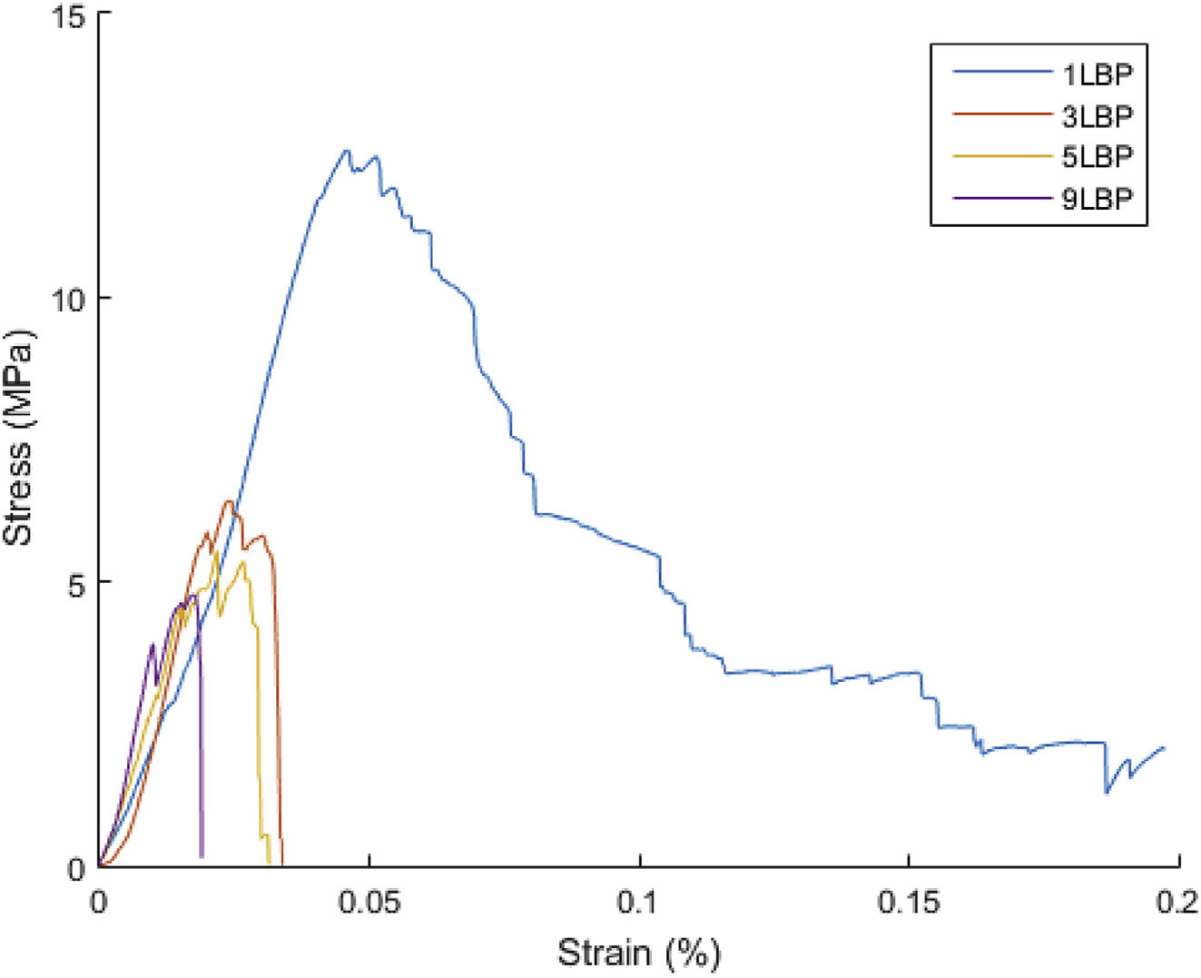
Stress-strain curves of each of the tested samples. While sample 1LBP shows high resistance with a progressive failure, other samples show reduced maximum strength with catastrophic failure events.

Examples of stress-strain curves from testing samples of each condition are shown in Fig. 2. Averaged values of mechanical properties extracted from such tests are summarised in table 1.

### 3.3 Microstructure and structure failure

**Fig. 3:**
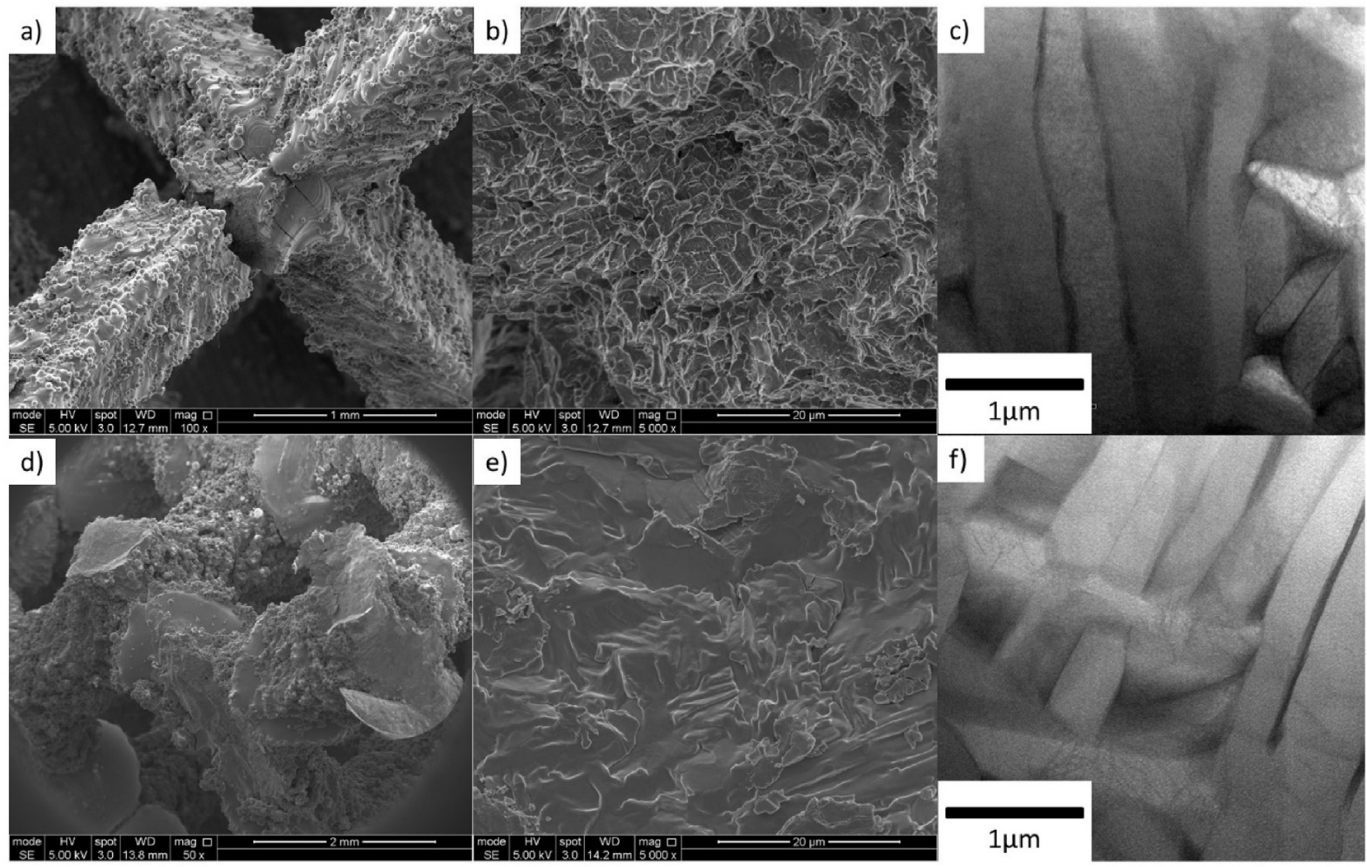
Microstructure and fractured areas by SEM and HREM. (a) Shows sample 1LBP fracture near the node. (b) Shows a magnified area of fractured face in (a) as a dimpled fracture. (c) Shows a fine α+β microstructure with a plate morphology of sub-1μm feature size. (d) Shows multiple fracture areas of sample 9LBP with a magnification in (e) showing a cleavage fracture due to material embrittlement. (f) Shows a similar α+β microstructure found in the sample melted multiple times.

Images of post-mortem compression samples are shown in Fig. 3. It can be seen that samples with 1 beam pass show fracture near the nodes of the lattice, see Fig. 3a. As reported previously [6], materials of this type are subjected to bending moments when displacement is applied, failing where the moments are greatest, hence, fracture is found in the vicinity of the nodes. High magnification imaging of the fracture shows a characteristic dimpled fractured area (Fig. 3b), the product of ductile fracture. On the other hand samples with the maximum number of beam passes show multiple fractured nodes aligned in a plane that contains a faceted fracture, see Fig. 3d. Observations at high magnification do not show dimples but rather the facets (Fig. 3e) characteristic of brittle fractures. This evidence is in agreement with earlier observations from the compression testing (Fig. 2), where samples with one beam pass showed progressive failure after the point of maximum resistance, and other samples presented a sudden failure driven by embrittlement. Therefore, a ductile-brittle transition in the fracture mode with increasing number of beam passes can be suggested.

### 3.4 Chemical analysis and X-ray diffraction

Results from chemical analysis of all materials are given in table 1. It can be seen that the composition of Ti–6Al–4V diamond lattices is affected drastically by the repeated heating passes, mainly through changes to the aluminium and oxygen content. While aluminium content reduces with increasing number of passes, oxygen content increases rapidly, beyond the 0.2 wt% upper limit set in standards for this alloy [7]. Still, the reduction in aluminium content does not result in the material falling out of tolerances (5.5–6.7 wt%).

**Fig. 4:**
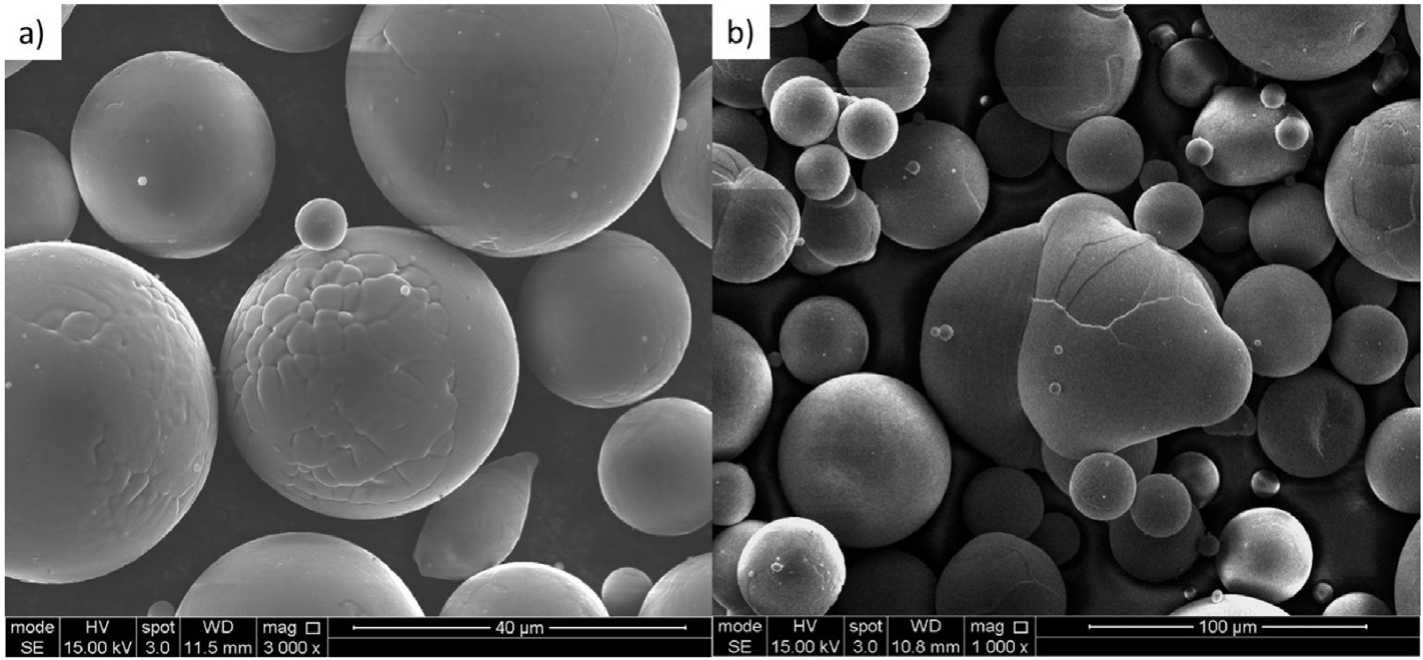
SEM images of prealloyed Ti–6Al–4V powder particles in: (a) virgin and (b) “discoloured” (post processing) condition.

**Fig. 5:**
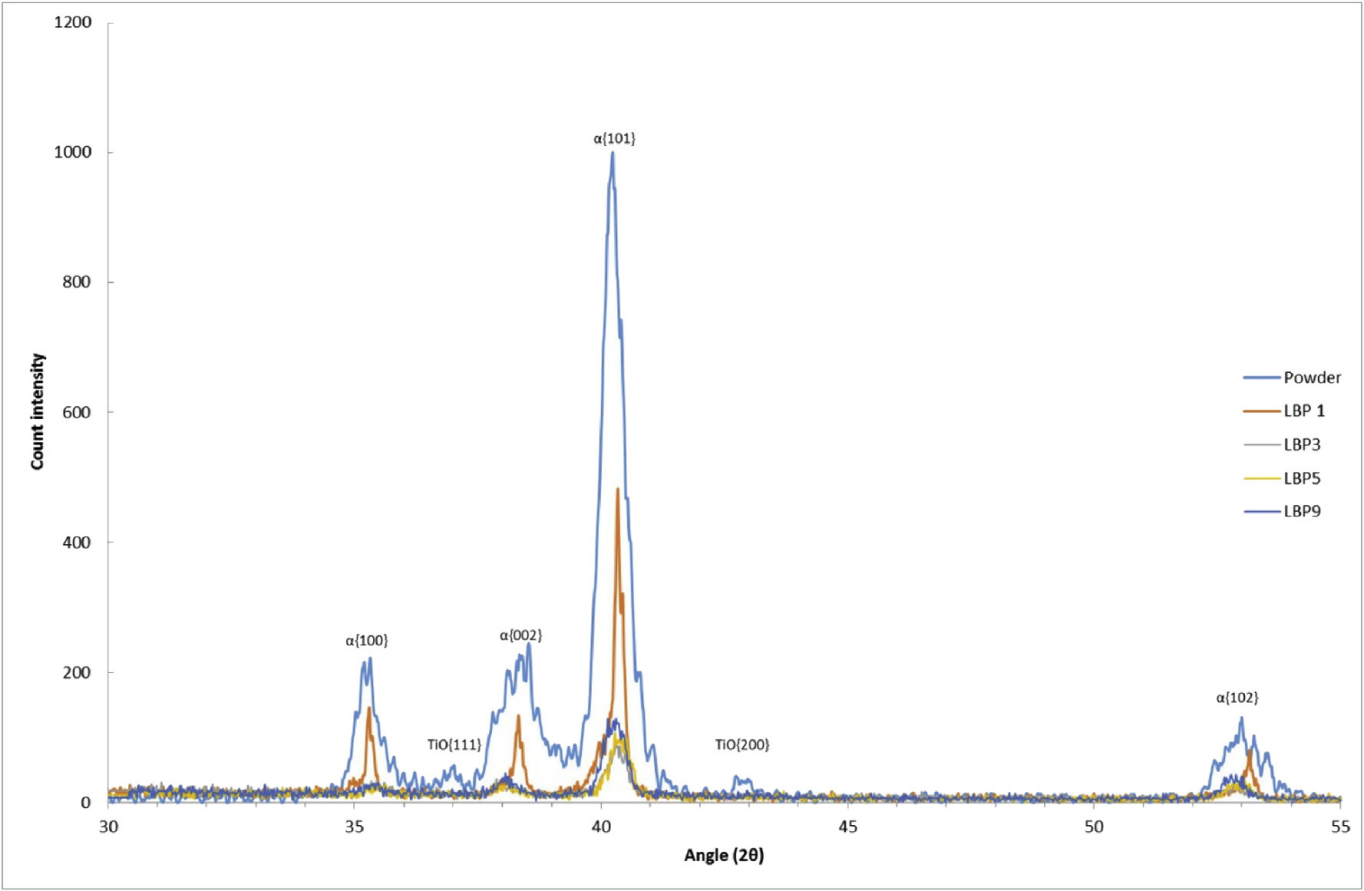
XRD patterns of diamond-like lattice samples and “discoloured” powder. While lattice samples show diffracted peaks in the alpha plane family, powder shows peaks of titanium oxide precipitates.

The oxygen content of powder not incorporated in to the lattices is also shown in table 1. Similar to the lattices, powder particles can be seriously affected by oxygen content, increased by being repeatedly heated, though never molten. An assessment was made of the powder moved to the sides of the powder-bed due to the effect of the argon recirculation flow. Such particles present a distinctive colouration from raw powder. Chemical analysis of these particles shows that they have similar oxygen content to the lattices melted with the maximum number of beam passes. High resolution imaging of virgin and discoloured Ti–6Al–4V particles, Fig. 4, showed surface areas partially modified in contrast to the virgin powders showing a “clean” morphology. Phase composition determined for all lattices and the powder by XRD (see Fig. 5) shows diffracted peaks related to cubic TiO and alpha titanium for the powder sample, while alpha titanium peaks only are seen for lattice samples.

## 4 Discussion

The principal hypothesis of this investigation was that a greater energy input in the Additive Manufacturing of Ti6Al4V, leading to a longer time spent in the molten state, would cause the material to dissolve larger amounts of oxygen form the chamber, and that this would be reflected in the mechanical properties of the materials produced. This can be confronted with the experimental results and is discussed along with secondary observations below.

### 4.1 Compositional change

**Fig. 6:**
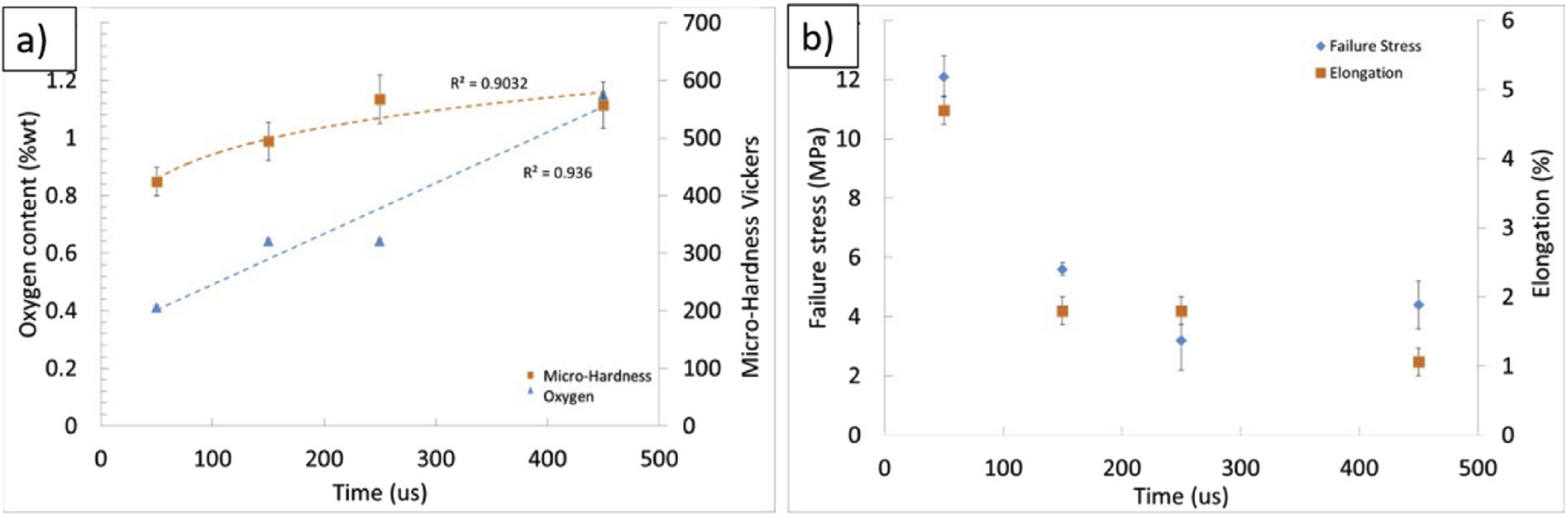
The trends in a) oxygen content and b) mechanical properties of the lattice materials with exposure time from number of laser beam passes.

Although the material was affected by the laser beam by a variable number of repeat passes, the energy input per unit length (*P* x *t*/*pd*) remained constant at all times (where *P* is beam power, *t* is beam exposure time and *pd* is the beam point distance), giving sufficient energy to provide melting conditions in the material. It is worth noting however that in doing this the material was in the molten state and exposed to the (only partially) inert atmospheric conditions for different times, proportional to the beam exposure time, multiplied by the number of laser passes. This quantity therefore represents an approximate time the material was in the liquid state, providing the most suitable conditions for oxygen pickup. The trend in oxygen content with this effective melting time is shown in Fig. 6a. As predicted, a trend for increasing oxygen with greater number of beam passes is observed, consistent, within experimental error, with a linear increase. Increasing the number of passes increases the total energy supplied, but will also increase the time the material spends at elevated temperature, when take up of any oxygen present is more likely, due to the increased kinetics. While it may be expected that a diffusion-limited process would show a non-linear trend, the high capacity of titanium for oxygen, in excess of the levels measured here, will mean that the process is limited by the kinetics of absorption (linear with time) rather than the distribution of oxygen within the metal. A similar effect on material hardness is found too, with a non-linear fitting, it suggested that a trend towards saturation in properties is due to compositional effects.

The compositional data show other effects. There are changes in the amount of aluminium in the alloy (Table 1), which can be related to the nature of the alloy and the conditions within the SLM process. Aluminium levels are likely to be reduced due to the higher volatility of this element (as seen for example in the higher vapour pressure of aluminium than titanium or vanadium); other AM systems, such as electron beam melting, also show a tendency toward the depletion of aluminium in this alloy, assisted in part by low partial pressures as the process is performed under vacuum [8].

Exploration of the powder that is not incorporated into the build shows high levels of oxygen (Table 1). This can be related to the way the laser and the powder particles interact. As the SLM system heats up with photons, whether a particle melts or not depends on the balance between how reflective or absorbing it is (affecting the thermal energy absorbed) and how thermally conductive the material is (affecting the dissipation of heat through the other powder particles it is in contact with). If melting occurs and the particle coalesces with others, it will contribute to the melt pool and the built component. If on the other hand the powder particle is ejected away by the beam, it is likely to be moved by the gas recirculation flow, and end up where the “discoloured” particles are found. Particles in the bed not exposed to the beam are unlikely to be disturbed or ejected by the recirculation flow and will not be moved. Particles that do get displaced therefore represent material which has been heated, but not melted and/or incorporated into the part. This heating is likely to be the stage in which the significant absorption of oxygen takes place.

### 4.2 Phase and microstructural development

It is interesting to note that none of the samples showed β-titanium peaks in XRD patterns, and that diffraction of the alpha phase showed the strongest diffraction from the {101} planes. This could be attributed to the high cooling rates generated in atomisation of raw materials and AM processing [9], favouring the presence of alpha phase. Previous reports have shown that AM processing of Ti6Al4V, with fast cooling rates, develops strong peaks corresponding to alpha phases, whereas slow cooling rates applied in post-processing heat treatments are more likely to result in beta phase [5]. Oxygen content on the other hand is observed in the form of TiO for powder samples only, suggesting that lattice materials have their oxygen content in solid solution, while the powder particles reach local oxygen concentrations (likely to be at the surface of the particles, bearing in mind that such particles have been heated, but probably not melted) which are sufficient to observe oxide as a phase. The presence of oxide and its thermodynamics of formation in SLM-AM is complicated, due to the high spatial and temporal variability in thermal conditions and the low partial pressure of oxygen, and this question would benefit from further investigation.

**Fig. 7:**
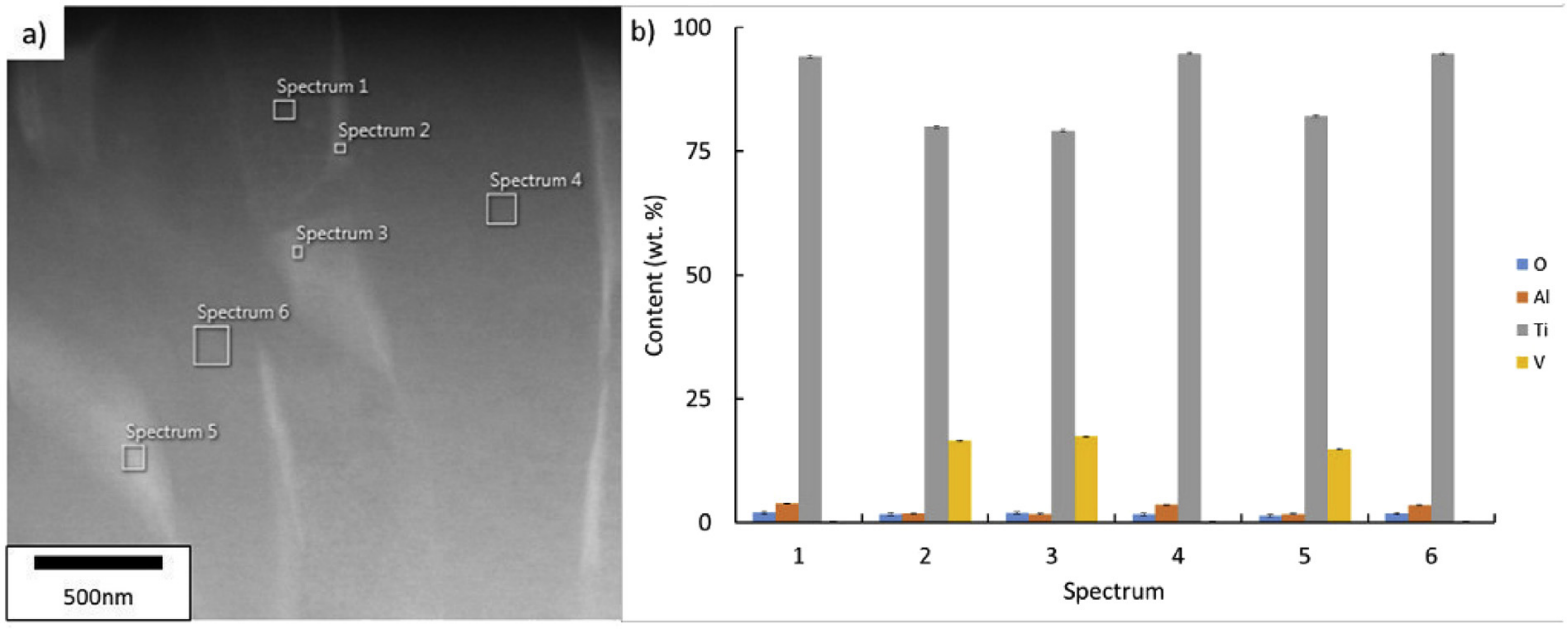
EDX analysis on sample 1LBP. (a) Lamellar α-β microstructure indicating areas of six spectra taken. (b) Normalised oxygen, aluminium, titanium and vanadium content in wt. % from all six spectra, error values are added for each element.

The microstructure of bulk laser AM Ti–6Al–4V is well reported in the literature [9, 10]. Usually subjected to high cooling rates, it is characterised by a fine lamellar α-β microstructure enclosed in columnar prior beta grains. In this work, a fine α-β lamellar structure is shown in all lattices with lamellae of a sub-1 μm width, see Figs. 3c and 3f. Further quantification of the alpha plate size, using the intersection method [11], found average values of 0.49 ± 0.18 μm and 0.55 ± 0.17 μm for 1LBP and 9LBP samples respectively, suggesting a similar microstructural feature size in both examples, and little variation in this with number of passes. Energy dispersive spectroscopy shows alternating α - β areas rich in their respective stabilising elements (see Fig. 7); for example, vanadium content increases in Ti-β areas and aluminium shows the same for Ti-α areas. No significant trend in oxygen distribution can be discerned with the sensitivity of EDX for light elements, although it is well known that the solubility of oxygen in titanium is significant, with increasing solubility in alpha phase up to 14.5% wt, and in solid solution will diffuse throughout the material [12]. It is therefore noteworthy that, although the samples show a clear trend in increasing oxygen levels with increasing number of beam passes, this is not reflected in a measurable change in the α-β microstructure.

### 4.3 Mechanical performance

As can be seen, the mechanical resistance does not increase with density as is usually reported for lattices, rather there is a decrease, also reflected in the elongation at which the point of maximum load occurred. However, hardness measurements suggest that strength levels for the metal (rather than the lattice structure formed from it) increase as the samples experience more laser beam passes. This points to there being a change in the properties of the Ti–6Al–4V, increasing its yield strength (hence raising the measured hardness under the approximate relation that hardness = 3 σ_y_ [13]), but reducing the strain to failure. In a lattice material, local areas, such as the regions around nodes where struts exposed to bending meet, will be exposed to higher strains than the nominal strain of the whole sample, and are locations where the conditions for brittle failure can rapidly be reached in materials prone to fracture. Failure at a lower overall strain will limit the stresses which the whole lattice can support, causing a reduction in the measured failure stress, as seen. This is supported by the SEM images at higher magnification, 3 (b) and (e) where the vicinity of the fracture is shown as a dimpled and cleavage fracture at low and high number of laser beam passes respectively.

As an alpha stabilizer, oxygen can be used to increase the strength in commercially pure (CP) titanium. A significant increase of this solute content however can lead to an unbalanced ratio of strength/ductility due to embrittlement [12]. In Ti–6Al–4V it has been found that structures with elevated oxygen levels higher increase, up to a point, their compressive strength. This result agrees qualitatively with previous results reported for materials processed by SLM, where material strength can appear to be higher when compared to other AM processes [14].

Due to titanium having a high reactivity with oxygen, pick-up for this interstitial element is likely each time the laser beam re-melts the material. Although SLM machines work under inert gas atmospheres, with oxygen sensors limiting oxygen to a maximum level of usually 1000ppm, and this work shows that oxygen pickup continues under these processing conditions. This allows oxygen pick up, which promotes hard and less ductile materials by Ti-α stabilisation. This factor should be taken into account in the selection of energy input into thin sections of any build (be it a lattice or other shape), and in the reuse and recycling of unmelted powder which has been exposed to elevated temperatures during processing. Although it is well known that strengthening in titanium alloys can be strongly related to grain size, in addition to solid solution effects [12], the microstructures found in samples 1LBP and 9LBP show a similar alpha plate size, implying that these processing conditions have similar cooling rates, and do not lead to significant size differences, meaning this cannot explain the effects observed.

**Fig. 8:**
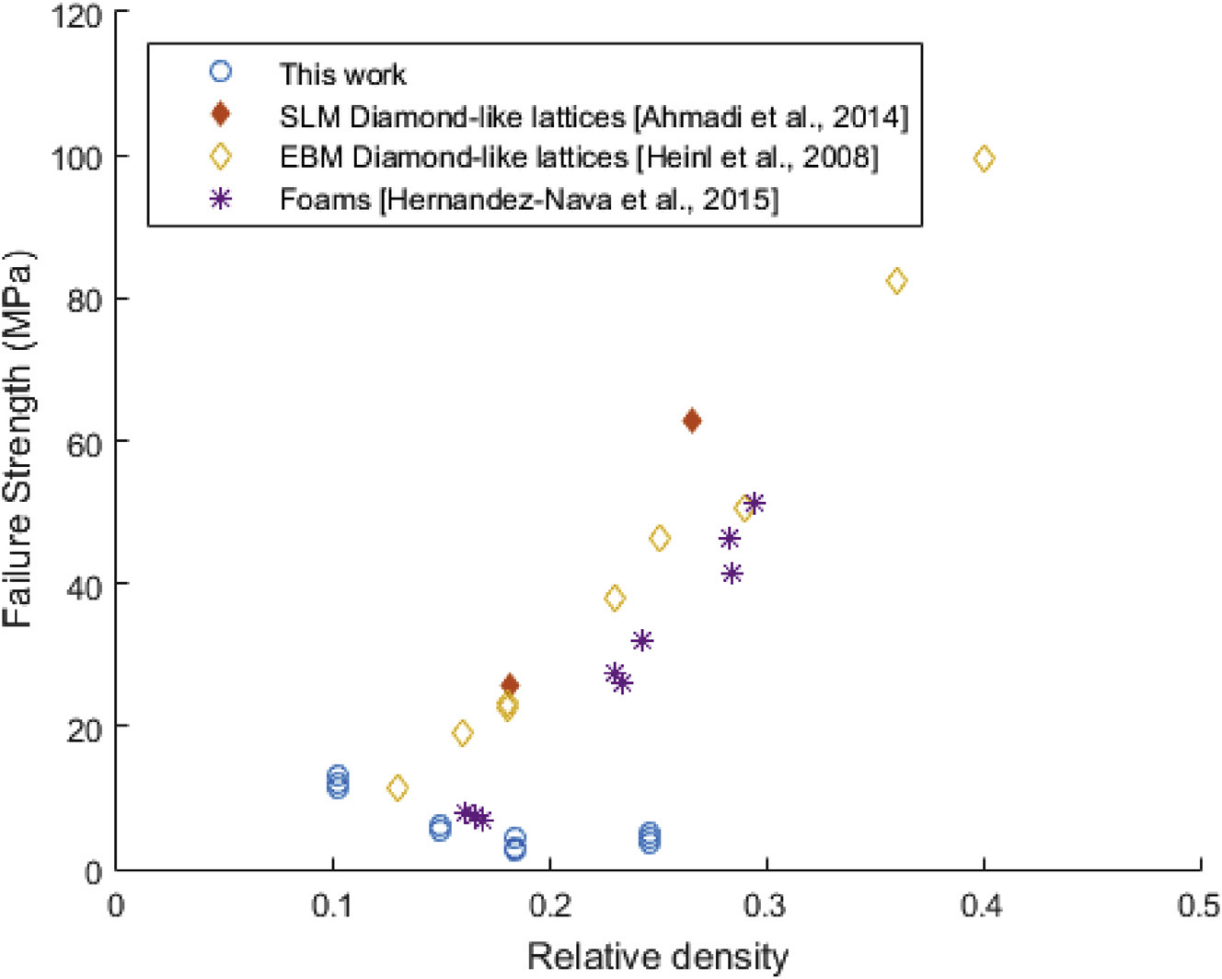
Failure strength with relative density of materials investigated in this work. Data from SEBM and SLM of diamond-like lattices and stochastic foams is added for comparison.

The mechanical response of all lattice materials can also be compared with cellular solids reported in the literature, see Fig. 8 where data by [15] and [16] are reported. In the chart, diamond-like lattice materials processed by SEBM and SLM are plotted in function of relative density and failure resistance. Also, data from metallic foams [17] is added for comparison. In the chart, it can be seen that diamond-like micro-truss materials tend to have an increasing mechanical resistance with higher density, as for foam structures. As reported in the literature [18], these materials present bending moments upon loading, failing eventually by plastic hinges at the node of the structure with an increase in failure strength with increasing density. Results of this type in such materials have been reported extensively [19]. However, the materials examined in this study show an opposite tendency. With a similar range of density (see Fig. 8), these tend to have a semi-inverse relationship. This can be attributed to the fact that in this work the samples have more differences than just the density, as discussed above. Taken with the observation of their increased hardness, the observed trend suggests embrittlement of the structure promoting an early collapse occurring from the stress strain curves, see Fig. 2.

## 5 Conclusions

Thin-walled materials in the form of diamond-like lattices were manufactured by selective laser melting successfully. Varying the number of repeated passes of the beam used in their processing leads to an increase in relative density, suggesting that extra material adhered to the lattice trusses. This implies that the additional thermal energy supplied at each set of beam coordinates led to melting more of the material. Characterised as cellular solids, the lattices show an opposite tendency to those reported in literature, either processed by SLM or EBM, with the more dense samples having less resistance under compression. Such behaviour is suggested to be due to material embrittlement due to oxygen pickup, and not to microstructural α-β modification of Ti–6Al–4V alloy. Comparisons with oxidised powder from the same process agreed with these observations. Overall, it is suggested that the atmospheric conditions within the AM machine expose the material to some level of oxygen continuously, unbalancing the strength/elongation ratio due to material embrittlement with more laser passes. In this work, the condition reached throughout the lattice materials is thought to be highly oxidised. This could be comparable to some regions of many parts that might be manufactured additively, oxidised depositions, overhangs in contact to anchor structures, etc. Understanding and characterising the properties and microstructures that result, as is done here, will aid the accurate modelling and prediction of real parts.

## Declarations

### Author contribution statement

Everth Hernández-Nava, Mauro Velasco-Castro: Conceived and designed the experiments; Performed the experiments; Analyzed and interpreted the data; Wrote the paper.

Ignacio Figueroa, Iain Todd: Contributed reagents, materials, analysis tools or data.

Russell Goodall: Conceived and designed the experiments; Analyzed and interpreted the data; Contributed reagents, materials, analysis tools or data.

### Funding statement

This work was supported by the EPSRC future manufacturing hub Manufacture using Advanced Powder Processes, MAPP (EP/P006566/1). MV-C would like to acknowledge funding from CONACyT through the scholarship 583061. EH-N would like to acknowledge funding from the Henry Royce Institute for Advanced Materials, funded through EPSRC grants EP/R00661X/1, EP/S019367/1, EP/P02470X/1 and EP/P025285/1. RG would like to acknowledge a Fellowship supported by the Royal Academy of Engineering under the RAEng/Leverhulme Trust Senior Research Fellowships scheme.

### Competing interest statement

The authors declare no conflict of interest.

### Additional information

No additional information is available for this paper.
